# The effect of high fiber snacks on digestive function and diet quality in a sample of school-age children

**DOI:** 10.1186/1475-2891-12-153

**Published:** 2013-11-25

**Authors:** Mary Brauchla, George P McCabe, Kevin B Miller, Sibylle Kranz

**Affiliations:** 1 Department of Nutrition Science, Purdue University, West Lafayette, Indiana 47907, USA; 2 Department of Statistics, Purdue University, West Lafayette, Indiana 47907, USA; 3 W.K. Kellogg Institute for Food and Nutrition, Battle Creek, Michigan 49017, USA

**Keywords:** Fiber, Whole grains, Diet quality, Constipation, Children

## Abstract

**Background:**

Dietary fiber (DF) intake in American children is suboptimal, increasing the risk of GI distress and contributing to poor diet quality. The objective of this study was to determine the effect of introducing two high-fiber snacks per day on gastrointestinal function as well as nutrient and food group intake in healthy children ages 7–11 years old.

**Methods:**

This study was a randomized controlled prospective intervention study of children 7–11 years of age (n = 81) attending a rural Midwestern elementary school. Children were randomized by classroom to consume two high-fiber snacks per day (total of 10-12 g DF) or their usual snacks for 8 weeks. Participants completed two 24-hour dietary recalls and a questionnaire about their GI health at baseline, mid-intervention (week 4), and post-intervention. Dietary data was entered into NDSR 2011 and t-tests utilized to assess changes. Analyses were completed in SAS 9.2.

**Results:**

Children consumed at least half their snack 94% of the time when a snack was chosen (89% of time). Participants in both the intervention and control group had healthy scores on the GI health questionnaire at all time points. The intervention group increased DF (P = 0.0138) and whole grain (WG) intake (P = 0.0010) at mid-intervention but after the intervention returned to their baseline DF intake (P = 0.2205) and decreased their WG intake (P = 0.0420) compared to baseline. Eating high-fiber snacks increased DF intake by 2.5 g per day (21% increase), suggesting displacement of other fiber-rich foods.

**Conclusions:**

Study results indicate that children accept high-fiber foods, thus making these high-fiber foods and snacks consistently available will increase DF intake.

## Background

Increasing dietary fiber (DF) intake was one objective of the 2010 Dietary Guidelines [1]. However, fiber intake in American children remains below recommended levels, with an average of 13.7 g/day in 6–11 year old children [2]. Suboptimal intake is especially concerning due to its association with poor diet and health outcomes, particularly in GI health [3,4].

Inadequate fiber intake in children is a risk factor for constipation [5], a serious public health concern with a prevalence of 3.2%-17.3% in American children [6]. Constipation involves painful physical symptoms that result in a lower quality of life [7,8]. Affected children have lower fiber intake than healthy children [5,9] and increasing bran and fiber intake can ameliorate symptoms [10]. The evidence for the association between fiber intake and constipation is robust and increasing dietary fiber is accepted as one of the first treatment recommendations [11].

DF-dense foods include fruits, vegetables and whole grains, which are underconsumed by American children [12]. Increased DF intake is also associated with better diet quality: young children with higher fiber consumption have more nutrient-dense diets because they consume more fruits, vegetables and grains [13] and older children with high fiber diets have been found to consume lower percentages of total and saturated fat [14].

In review of this research there is a clear need to increase DF consumption in children to improve their diet quality and lower the risk for constipation and chronic disease. This study was a cluster randomized-controlled prospective community-based intervention conducted to determine the effect of introducing two high-fiber snacks per day on gastrointestinal function as well as nutrient and food group intake in healthy children ages 7–11 years old.

## Methods

This study was an 8-week long, cluster randomized, controlled community-based intervention in 7–11 year old children at one elementary school. Teachers were solicited for interest in the study and 11 allowed their students to participate. Classrooms were paired by number of participants and one of each pair was randomly selected to participate in the intervention. The protocol was approved by the Institutional Review Board of Purdue University and written informed consent was obtained from children and their parents before taking part in the study. Children with digestive disorders, food restrictions and food allergies were excluded from the study.

Upon recruitment, participants provided data on demographics, gastrointestinal function, and usual diet (baseline), followed by an 8-week long intervention period during which the intervention group was instructed to consume two high-fiber snacks per day (7 days per week) while the control group consumed their usual snacks. At the midpoint of the intervention (week 4), a second set of data was collected on gastrointestinal function and usual dietary intake. After the eighth and final week of the intervention, data on gastrointestinal function and usual dietary intake were collected again (post-intervention). The number of participants recruited was based on a sample size calculation assuming 80% power, 0.05 error and a confidence interval of 12 g of fiber consumed, which would double the amount of fiber consumed per 1000 kcals as seen in NHANES [3].

To encourage children’s snack consumption, a choice of two high-fiber snacks (matched to be within 1 g fiber) was offered at each eating occasion. The snacks were chosen for use in the study based on their fiber and energy content so that the combined fiber provided by the study snacks was 10-12 g per day, with the goal of increasing fiber consumption by 8 g per day. The snacks contained on average 157 kcals and 5.1 g of fiber; consuming all of both snacks would result in an intake of 314 kcals and 10.2 g of fiber on average. All study foods were unpacked to prevent brand recognition and packaged and labeled by the research staff with the date and eating occasion. In addition, each child was also offered an 8-ounce carton of skim milk at each snack occasion to provide fluids to prevent GI distress. The researchers served the snacks and recorded each child's snack consumption as ‘none’, ‘one-quarter’, ‘one-half’, ‘three-quarters’, or ‘all’. The list of snacks offered is shown in Additional file 1: Table S1.

Snacktimes were scheduled at the most convenient time for teachers and students; however, due to time demands on teachers it was not always possible for researchers to serve the snacks twice a day in each classroom. Two classrooms could only accommodate one snack occasion per day so children in these classrooms (n = 15) were given their afternoon snacks to take home and followed the same procedures utilized for weekend snacks. To maintain snacks over the weekend, each Friday children received a labeled paper bag with two options for each snack occasion with snacks labeled for the day and time (AM or PM) for each weekend day. Children and parents were instructed to return all uneaten portions or the empty snack bags to the research staff in school on the next school day.

At baseline, parents completed a one-page survey providing information on children's demographic characteristics. The survey was a truncated version of the demographic data collection survey tool of the National Health and Nutrition Examination Survey (NHANES) administered by the Centers for Disease Control and Prevention (CDC).

Surveys on gastrointestinal health were collected at three time points: baseline, mid-intervention, and post-intervention. At these times, children and parents were asked to complete an 8-question Regularity Questionnaire about the child’s digestive health (Additional file 2: Table S2) that included the Bristol Stool Chart developed by Lewis et al. [15].

To assess children’s total daily food intake all participants completed two 24-hour recalls via telephone at baseline, mid-intervention, and post-intervention. One recall was taken for a Monday-Thursday representing weekday intake and a second taken between Friday-Sunday, representing a weekend day intake. While many studies include two weekday and one weekend recall, only two recalls were utilized in this study to minimize participant burden and to provide a comparison to NHANES data. Both recalls were completed within 10 days and parent assistance was encouraged for younger participants, per NHANES procedures [16]. Previously, research has indicated that self-reported intakes from children in this age group are reliable and align with parent reports [17-19], although younger children were still encouraged to seek parental assistance when needed.

Each recall was concluded with the question of whether the reported intake had been reflective of usual food intake or not, for instance due to illness; if not representative of usual intake, the recall was coded as unreliable. Other recalls were coded as unreliable if the child could not recall one or more foods consumed or the food amount. All recalls were conducted exclusively via telephone by trained researchers at the Purdue University, Department of Nutrition Sciences using the standardized methodology provided by the Nutrient Data System for Research (NDSR) version 2011, developed by the Nutrition Coordinating Center (NCC), University of Minnesota, Minneapolis, MN. Upon training all researchers in the specific recall methods, a mock 24-hour recall was conducted and inter-interviewer reliability tested. Results showed that interviewer reliability was within 5%.

When both recalls were completed, the information was averaged and the two-day average was used in the analysis to represent usual intake at that time period. If only one recall was completed, information from that one recall was used (6% of the observations). Total daily energy intakes of below 500 kcals or above 3,500 kcals were considered implausible intake reports and their accuracy confirmed with the parents via a follow-up phone call.

A total of 81 children consented and began the study. One child dropped out due to moving out of the area, and the remaining 80 children completed the study. Participants’ demographic characteristics are represented in Table 1.

**Table 1:** Participant demographics

	**Intervention (n = 40)**		**Control (n = 41)**	
**Age groups**	**7-8**	**9-11**	**7-8**	**9-11**
**Total participants**	8	32	4	37
Male	5	16	2	20
Female	3	16	2	17
**Ethnic group**^**1**^				
Asian	0	3	0	0
African American	1	1	0	1
Hispanic/Latino	1	2	2	4
White	5	21	2	23
Other	1	1	0	2

^1^Information on ethnic group was not available for some subjects.

### Statistical analysis

All data were entered into Excel data spreadsheets using double-entry procedures. In short, all data were entered by two different researchers and checked by a third researcher using a program code that overlays the spreadsheets and produces an error message for each cell that has non-corresponding content. Upon correction of the data, the matching procedure was repeated and no additional error messages were produced. The data were subsequently transferred into SAS version 9.2 for statistical analysis (SAS Institute Inc., Cary, NC, USA). Descriptive statistics, means, standard deviations, and proportions were generated.

In total, 22 dietary recalls (5%) were excluded from the analysis as they were deemed unreliable or were not representative of usual intake: 7 recalls were excluded from baseline, 5 recalls were excluded from mid-intervention and 6 recalls were excluded from post-intervention. Eleven of these recalls were excluded because the report was not representative of the child’s usual intake (the child skipped a meal, ate much more than usual, was ill, etc.). Descriptive statistics and test of comparisons (student’s t-test) were performed on total energy, food group, and nutrient data to discern statistical differences between the intakes of children in the intervention and the control group as well as changes within groups but between time points (assessed with paired t-tests).

Questions 1–6, 8, and 9 of the Regularity Questionnaire were coded on a 5-point Likert scale to explore the child’s digestive health symptoms with 1 representing ‘constipated’ and 5 being ‘healthy’. Due to the high collinearity of the children’s reported responses to the first six questions, the scores were added to create a cumulative score. Questions 7–9 were evaluated individually. Since the original design of the Bristol Stool Chart was based on 7 response options [15], question seven was coded on a 7-point scale. Missing values for any question remained coded as ‘missing’. Statistical analyses assessing the difference ratings of digestive health between children in the control and the intervention group were assessed using a student’s t-tests while changes within groups but over time were evaluated with a paired student’s t-tests.

Missing amounts for snack consumption comprised less than 1% of first snack observations and missing values were imputed using the sample’s average snack consumption amount for that snack (between ‘3/4 consumed’ or ‘all consumed’ for all snacks). Data from five children were excluded from the analysis, as they did not choose to eat a snack on at least 50% of the eating occasions; two of these children declined snacks for reasons unrelated to study procedures. Snack preference was assessed with a general linearized model and adjusted for age and gender.

## Results and discussion

Total energy and nutrient intakes for both groups at each time point are shown in Table 2. There were no significant differences in total energy intake at baseline between the groups. At mid-intervention there was a nonsignificant increase in calories from 1529 at baseline to 1572 (P = 0.3073) in the intervention group. At baseline the intervention group had a higher intake of total dietary fiber (12.03 ± 4.39 g) than the control group (10.10 ± 4.42 g; P = 0.0347), which also resulted in a higher fiber density intake for the intervention group (8.00 ± 2.53 g) compared to the control group (7.09 ± 1.79 g; P = 0.0429). Since children were not individually randomized but by group (on the basis of their home classroom), thus, n = 24 of the intervention participants attended a classroom that was at an accelerated level, which might have reflected shared characteristics within that group. In fact, the students in this classroom had an average baseline fiber intake of 12.32 g at baseline. There were no other differences in macro- or micronutrient intake between groups at baseline. At the mid-intervention timepoint, the intervention group increased their intakes of total dietary fiber (14.58 ± 5.06 g; P = 0.0138), insoluble dietary fiber (9.63 ± 3.21 g; P = 0.0026), and fiber density (9.49 ±2.80 g; P = 0.0081) while the control group had no change in macro- or micronutrient intakes at mid-intervention compared to baseline. However, at the post-intervention timepoint all macro- and micronutrients had returned to baseline values in the intervention group.

**Table 2:** Total energy, macro, micro-nutrient intake by group at baseline, mid-intervention, and post-intervention by group (mean, SD)

		**Intervention**			**Control**	
	**Baseline**	**Mid-intervention**	**Post-intervention**	**Baseline**	**Mid-intervention**	**Post-intervention**
**Number of participants**	34	33	32	37	36	35
**Number of recalls**	63	64	61	69	68	67
**Energy**	1528.65	1571.88*	1525.56	1408.78	1401.13	1385.60
**(kcal)**	(369.14)	(412.71)	(382.65)	(493.38)	(409.46)	(376.02)
**Total fat**	56.22	54.49	56.93	51.60	50.81	50.09
**(g)**	(18.42)	(22.50)	(17.13)	(19.99)	(15.95)	(18.54)
**Total carbohydrate (g)**	205.00	215.23*	196.62	187.81	187.85	185.36
	(58.92)	(55.88)	(57.28)	(69.65)	(63.48)	(53.55)
**Total protein**	56.32	61.72*	60.21*	52.84	52.62	51.74
**(g)**	(14.16)	(19.67)	(19.99)	(19.60)	(19.67)	(17.19)
**Cholesterol**	146.46	163.07	186.38*	136.86	142.25	137.91
**(mg)**	(72.93)	(148.65)	(128.84)	(70.16)	(72.34)	(83.26)
**Total saturated fatty acids (SFA) (g)**	19.55	19.23	20.15	19.23	17.93	18.20
	(6.90)	(9.15)	(6.86)	(8.29)	(6.39)	(8.24)
**Total dietary**	12.03*	14.58*†	11.06	10.10	9.99	10.00
**Fiber (g)**	(4.39)	(5.06)	(4.73)	(4.42)	(4.02)	(11.06)
**Soluble dietary**	4.37	4.88*	4.13	3.44	3.23	3.62
**Fiber (g)**	(3.03)	(2.16)	(2.22)	(2.17)	(1.19)	(1.75)
**Insoluble dietary fiber (g)**	7.61	9.63*†	6.87	6.65	6.70	6.29
	(2.50)	(3.21)	(3.26)	(2.89)	(3.11)	(2.86)
**Fiber density**	8.00*	9.49*†	7.32	7.09	7.11	7.19
**(g/1000 kcals)**	(2.53)	(2.80)	(2.96)	(1.79)	(2.18)	(2.55)
**Calcium**	848.32	953.13	907.97	868.46	829.47	771.33
**(mg)**	(340.05)	(398.33)	(336.79)	(378.11)	(383.04)	(353.85)
**% calories from fat**	32.20	29.68†	32.98	31.90	32.04	31.47
	(6.82)	(6.95)	(6.31)	(4.75)	(5.31)	(6.02)
**% calories from carbohydrate**	52.62	54.41	50.77	52.79	52.39	53.33
	(7.50)	(8.32)	(7.43)	(6.65)	(6.40)	(7.76)
**% calories from protein**	15.22	15.94	16.28	15.37	15.62	15.23
	(3.56)	(3.33)	(3.93)	(3.63)	(4.14)	(3.44)
**Added sugars**	59.47	61.40	53.48	53.29	57.27	50.59
**(g)**	(30.84)	(37.90)	(30.55)	(30.26)	(34.80)	(28.62)

*Intervention greater than control (p < 0.05).

^†^Significant from baseline (p < 0.05).

Food group serving intake by food group and time point is shown in Table 3. At baseline, the intervention group had a higher intake of whole grains (1.15 ± 1.02 g; P = 0.0013) compared to the control group (0.51 ± 0.68 g) as well as a higher intake of grains (6.11 ± 2.38 g) compared to the control group (5.12 ± 2.28 g; P = 0.0399). Intake of whole grain in the intervention group was significantly higher at the mid-intervention time point (2.08 ± 1.39 g) than baseline (P = 0.0010), but at post-intervention (0.77 ± 1.00 g) decreased below both mid-intervention and baseline levels (P = 0.0001 and P = 0.0420, respectively). However, intake of grains did not increase in the intervention group at mid-intervention (6.86 ± 1.77 g) compared to baseline (P = 0.110), but still remained higher than intake in the control group (5.08 ± 1.95 g) (P = 0.0001).

**Table 3:** Food group intake in servings at baseline, mid-intervention and post-intervention by group (SD)

		**Intervention**			**Control**	
	**Baseline**	**Mid-intervention**	**Post-intervention**	**Baseline**	**Mid-intervention**	**Post-intervention**
**Number of participants**	34	33	32	37	36	35
**Number of recalls**	63	64	61	69	68	67
**Fruits**	1.04 (1.20)	0.97 (1.03)	0.98 (1.15)	0.92 (0.93)	0.95 (1.06)	1.01 (1.02)
**Vegetables**	1.15 (1.26)	1.05 (0.60)	1.06 (0.82)	1.33 (0.75)	1.18 (1.14)	1.25 (0.90)
**Grains**	6.11 (2.38)*	6.86 (1.77)*	6.30 (2.28)*	5.12 (2.28)	5.08 (1.95)	5.45 (1.58)
**Whole grains**	1.15 (1.02)*	2.08 (1.39)*†	0.77 (1.00)†	0.51 (0.68)	0.59 (0.81)	0.56 (0.76)
**Protein**	3.45 (1.80)	3.39 (1.97)	3.46 (2.16)	2.88 (1.80)	2.96 (1.79)	3.03 (1.82)
**Dairy**	2.17 (1.02)	2.22 (1.21)	2.25 (1.18)	2.25 (1.16)	2.13 (0.97)	1.99 (1.40)
**Fats**	1.22 (1.21)	1.35 (1.13)	1.92 (1.67)†	1.65 (1.57)	1.35 (1.11)	1.47 (1.22)
**Sweets**	0.44 (0.54)	0.55 (0.68)	0.55 (0.64)*	0.50 (0.71)	0.50 (0.55)	0.33 (0.37)
**Beverages**	1.57 (1.02)	1.22 (0.79)	1.47 (1.20)	1.21 (0.84)	1.23 (0.96)	1.32 (1.03)

*Intervention greater than control (p < 0.05).

^†^Significant from baseline (p < 0.05).

Average responses to the Regularity Questionnaire did not change over time; both intervention and control groups had healthy scores at baseline and scores in both groups remained healthy over the course of the study.

A total of 3,605 snacks were selected for consumption over the 8 week intervention, with an average of 106 snacks chosen per participant. Children chose cereal over crackers (P < 0.0001) and bread (P < 0.0001), and chose bread over crackers (P < 0.0001). When covariates were included, age group was significant in preference of bread to crackers (P = 0.0134), and gender was significant in preference of cereal to bread (P = 0.0298). Including all helpings of snacks, children chose cereal 54% of the time, bread 30% of the time and crackers 16% of the time as shown in Table 4.

**Table 4:** Summary of snack consumption and choice (%)

	**All eating occasions**
**Cereal chosen**	1936 (54%)
**Crackers chosen**	570 (16%)
**Bread chosen**	1099 (30%)
	**Consumption of first snack**
**Snack chosen but not consumed**	18 (<1%)
**¼ of snack consumed**	211 (6%)
**½ of snack consumed**	483 (14%)
**¾ of snack consumed**	415 (12%)
**All of snack consumed**	2408 (68%)

Compliance with snack consumption was excellent for most participants as they chose at least one snack at 89% of snack occasions. When participants chose a snack, they consumed at least half of the snack over 94% of the time, with children consuming the entire snack 68% of the time. However, there were 5 children who did not consume their snacks on a regular basis. Children chose a second snack helping (either the same snack as originally chosen or the other option) at 501 (14%) of eating occasions.

No adverse events were reported during this study.

## Discussion

The aim of this study was to evaluate changes in nutrient and food group intake with the addition of high-fiber snacks in children ages 7–11 years old. Overall, results indicated that children accepted the snack foods easily and significantly increased their fiber intake during the study. This is particularly noteworthy as other sources of dietary fiber, such as fruits, vegetables and WG, were consumed at levels below recommendations, as consistent with previous data [1,20,21].

Surprisingly, we observed few differences in macro- and micronutrient intake during this study. No significant difference in dairy or calcium intake was seen at the mid-intervention timepoint in the intervention group even though children in the intervention group were served skim milk twice per day with their snacks. Intake of grains and calories did not increase significantly at mid-intervention in the intervention group although the snacks were all grain-based and the increased consumption in grains fell just short of statistical significance. This is noteworthy as the majority of participants in the intervention group (n = 26) were in classrooms that did not usually allow snacks, so the intervention added two eating occasions to their usual routine. While total grain intake did not change, whole grain intake increased significantly during the intervention in the intervention group only indicating that the children were likely displacing refined grains with whole grains over the remainder of the day. This is a desirable change toward the recommendations of the Dietary Guidelines for 2010; however, children’s intake still fell short of the recommendation of consuming half their grains as whole grains. Due to the high compliance in consuming the high-fiber snacks one would expect the fiber intake at mid-intervention to have been much higher in the intervention group, but the increase in total daily dietary fiber was only 2.2 g – again, this is likely due to displacement of other foods in the diet over the remainder of the day, in particular replacing refined grains with whole grains. In addition, added sugars intake did not increase in the intervention group at mid-intervention even though the intervention foods included sweetened cereals, which were the most popular snack choice. Overall, these results suggest that participants modified intake, compensating for the added sugar content in the foods and beverages consumed during the rest of the day. No systematic change in macronutrient intake was observed, thus, consuming the study snacks did not result in uniform displacement of any particular food group or foods.

Although the intervention group only increased their total average dietary fiber intake by 2.2 g during the intervention, these results indicate that children’s fiber intake can be increased by offering high-fiber foods. To date, it is unknown if/where dietary fiber consumption is at its maximum (the ceiling of intake), from which point on it would be likely that spontaneous displacement of other fiber-rich foods may occur. Our results show that although fiber intake increased significantly, total energy intake remained stable. Thus, we successfully increased dietary fiber density in this population. The need to increase fiber intake in the diets of children without additional calories could be met by offering high-fiber foods at most or even all eating occasions. It is also noteworthy that even with an increase in fiber intake there was no increase in undesirable digestive symptoms (e.g. flatus, abdominal pain), which are often a reason for avoiding high fiber foods. The results of this study indicate that small increases in fiber have no adverse gastrointestinal side effects in children. Since this study was only based on two snacks per day, the potential for increasing overall daily dietary fiber intake to approximate the dietary intake guidance recommendation is supported; however, future studies are needed to provide additional evidence. In addition, fiber intake in the intervention group returned to baseline values post-intervention, which is consistent with previous research indicating that individuals will alter fiber intake during a study but return to baseline levels after completing the intervention [22].

Scores on the Regularity Questionnaire suggest that the majority of children had healthy gastrointestinal health at each time point. However, because this sample had such high scores at baseline, the effect of the intervention was difficult to evaluate. The intervention time of 8 weeks may also have been too short to discern differences in the effects of fiber on GI health. These results do indicate that children were comfortable completing the survey as over 80% of children returned their surveys at each time point. Therefore, future research could include this survey within a longer intervention trial that specifically targets children with symptoms of GI distress.

The high fiber cereals offered were the most popular snack food choices, followed by bread and crackers. However, the high compliance rate indicated that children accepted snacks in all 3 categories, which should be noted when designing future fiber interventions. Overall, the results suggest that children will accept high-fiber snacks over a long time period, with only 5 of 40 children consistently refusing to eat any snacks. It should be noted that 3 of these children were in the same classroom which had only 5 participants and their noncompliance may have resulted from discomfort in eating snacks in front of their classmates, who were not allowed to eat during that time.

This study has several strengths and limitations which must be acknowledged. The sample was limited to children in rural Indiana, and therefore results cannot be generalized to the American population. The procedures included one weekday and weekend recall instead of the two weekday and one weekend recall utilized in other countries. In addition, NDSR 2011 does not categorize dietary fiber from each food group, so researchers cannot distinguish between fiber from fruits, vegetables and whole grains. During the 8-week intervention period, compliance and acceptance of high-fiber snacks was very high; however, further research with a longer time period is needed to evaluate whether children could continue to accept high-fiber snacks over time or if a “tiring” effect might be evident. Utilizing high-fiber fruits and vegetables in combination with grain-based foods should be considered in future studies as more variety may increase children's interest in eating the study foods. In addition, the healthy GI scores at baseline prevented evaluation of how this type of intervention would affect symptoms of GI distress: future research should focus on children who are at risk for gastrointestinal problems.

## Conclusions

Results of this study suggest that children will accept and eat high-fiber snacks and that they will increase their total dietary fiber as long as the snacks are offered but return to their usual diet once the high-fiber snacks are no longer provided. This observation indicates the need to target school-feeding as an effective venue to improve child nutrition. Studies targeting families to change children's diets might not be successful, as families in this study did not continue feeding the children high-fiber snacks. Although no formal data collection was conducted, the children liked the snack foods and indicated to the researchers that they looked forward to the snack time in school. However, many parents did not agree to purchase and provide high-fiber snacks in the child's home environment. Therefore, if schools were to offer high-fiber foods consistently during the school year, children would be more likely to increase their fiber intake and diet quality at least during the academic portion of the year.

Future studies should incorporate incentives for parents to change the foods provided to children at home. In addition, nutrition education and food purchasing strategies to make the inclusion of more fruits, vegetables and whole grains with high-fiber feasible for families might be beneficial.

## Competing interest

The authors declare that they have no competing interests. The authors thank the student volunteers who assisted with this study.

## Authors’ contributions

The study was designed and guided by SK and KM; MB oversaw and conducted the data collection and GM performed the statistical analysis; MB wrote the manuscript draft, and all authors provided crucial feedback on the final version; SK had final responsibility for the manuscripts. All authors read and approved the final manuscript.

## Supplementary Material

Additional file 1: Table S1 Snack list. Click here for file

Additional file 2: Table S2 Child Regularity Questionnaire. Click here for file
