# Supplementary material for: The effect of high fiber snacks on digestive function and diet quality in a sample of school-age children
Source: Nutr J. 2013 Nov 25;12:153. doi: 10.1186/1475-2891-12-153 (PMC4222504; doi:10.1186/1475-2891-12-153)
Supplement: Additional file 1: Table S1 — Snack list. [file 1475-2891-12-153-S1.docx]

**Supplemental File 1. Snack list**

|  | **Cereals** |
| --- | --- |
| **1** | Kellogg’s Frosted Mini-Wheats Little Bites Chocolate® |
| **2** | Kellogg’s FiberPlus Cinnamon Oat Crunch® |
| **3** | Kellogg’s Frosted Mini-Wheats Little Bites Original® |
| **4** | Kellogg’s Cracklin Oat Bran® |
| **5** | Kellogg’s Frosted Mini-Wheats Bite Size Blueberry Muffin® |
| **6** | Kellogg’s FiberPlus Berry Yogurt Crunch® |
| **7** | Kellogg’s NutriGrain Bar® |
| **8** | Kellogg’s Frosted Mini-Wheats Big Bite® |
| **9** | Kellogg’s Frosted Mini-Wheats Blueberry® |
| **10** | Kellogg’s Frosted Mini-Wheats Strawberry® |
| **11** | Kellogg’s Frosted Mini-Wheats Touch of Fruit in the Middle Mixed Berry® |
|  | **Crackers** |
| **12** | Kellogg’s Special K Snack Crackers - Savory Herb® |
| **13** | Kellogg’s Special K Crackers Multigrain® |
| **14** | Kellogg’s All-Bran Crackers Multi Grain® |
|  | **Breads** |
| **15** | Sara Lee Soft and Smooth White Bread with Whole Wheat® |
| **16** | Sara Lee Soft and Smooth 100% White bread with Ca/Vit D® |
| **17** | Pepperidge Farm Deli Flats (Whole Grain White)® |
| **18** | Thomas 100% Whole Wheat Bagel Thins® |
| **19** | Pepperidge Farm Stone Ground Whole Grain Bread® |
| **20** | Thomas 100% Whole Wheat Mini Bagel® |
| **21** | Arnold Whole Wheat Bread® |
| **22** | Arnold Sandwich Thins® |
| **23** | Thomas Light Multi-Grain English Muffin® |
| **24** | Thomas Plain Bagel Thins® |
