# Supplementary material for: The effect of high fiber snacks on digestive function and diet quality in a sample of school-age children
Source: Nutr J. 2013 Nov 25;12:153. doi: 10.1186/1475-2891-12-153 (PMC4222504; doi:10.1186/1475-2891-12-153)
Supplement: Additional file 2: Table S2 — Child Regularity Questionnaire. [file 1475-2891-12-153-S2.docx]

**Supplemental File 2: Child Regularity Questionnaire**

**Please complete this questionnaire by picking the best answer for the following questions:**

1. In the past week, how often did you feel puffy and uncomfortable in the belly?

- Never
- Almost Never
- Sometimes
- Often
- Almost Always

2. In the past week, how often did you strain or squeeze to try and pass a poop?

- Never
- Almost Never
- Sometimes
- Often
- Almost Always

3. In the past week, how often did you finish pooping but it still felt like there was some poop that didn’t come out?

- Never
- Almost Never
- Sometimes
- Often
- Almost Always

4. In the past week, how often did you have gas (fart)?

- Never
- Almost Never
- Sometimes
- Often
- Almost Always

5. In the past week, how often did you feel discomfort or hurt in your tummy below your belly button?

- Never
- Almost Never
- Sometimes
- Often
- Almost Always

6. In the past week, how often did you poop?

- Never
- Almost Never
- Sometimes
- Often
- Almost Always

7. If you look at the picture chart attached, which pictures look like your poop did most often this past week?

(use attached picture chart)

- Type 1
- Type 2
- Type 3
- Type 4
- Type 5
- Type 6
- Type 7
- I did not look at my poop last week


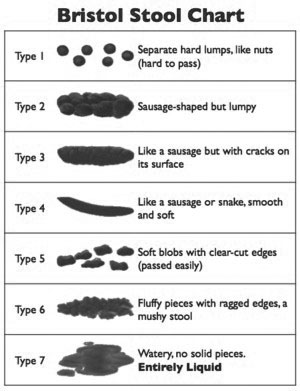


8. Did you miss school or playtime because of problems with your tummy or wanting or needing to poop?

- - Never
  - Less than 3 times this week
  - More than 3 times this week
  - At least once a day

9. What size is your poop closest to?

- - Grape
  - Golf ball
  - Baseball
  - Softball
